# Radioiodine-131 Therapy Used for Differentiated Thyroid Cancer Can Impair Titanium Dental Implants: An In Vitro Analysis

**DOI:** 10.3390/cancers15092558

**Published:** 2023-04-29

**Authors:** Doina Piciu, Simion Bran, Marioara Moldovan, Simona Varvara, Andra Piciu, Stanca Cuc, Cristina Moisescu-Goia, Elena Barbus, Alexandru Mester, Florin Onisor

**Affiliations:** 1 Department of Endocrine Tumors and Nuclear Medicine, Institute of Oncology “Ion Chiricuta”, 400015 Cluj-Napoca, Romania; 2 Doctoral School, University of Medicine and Pharmacy “Iuliu Hatieganu”, 400012 Cluj-Napoca, Romania; 3 Department of Maxillofacial Surgery and Implantology, University of Medicine and Pharmacy “Iuliu Hatieganu”, 400012 Cluj-Napoca, Romania; 4 Institute of Chemistry “Raluca Ripan”, University Babes-Bolyai, 400294 Cluj-Napoca, Romania; 5 Faculty of Exact Sciences and Engineering, “1 Decembrie 1918” Alba Iulia University, 510009 Alba Iulia, Romania; 6 Department of Medical Oncology, University of Medicine and Pharmacy “Iuliu Hatieganu”, 400012 Cluj-Napoca, Romania; 7 Department of Oral Health, University of Medicine and Pharmacy “Iuliu Hatieganu”, 400012 Cluj-Napoca, Romania

**Keywords:** titanium implant, osseointegration, radioiodine-131, differentiated thyroid cancer

## Abstract

**Simple Summary:**

The alterations on dental implants seen after therapeutic radioiodine-131 exposure have important clinical impact. Several microstructural alterations start to appear at 24 h up to 2 weeks after exposure; this affects the pores walls which tend to be eroded.

**Abstract:**

Background: The aim was to assess, in vitro, the effects of radioiodine-131 (I-131) on the structure of titanium implants. Material and Methods: A total of 28 titanium implants were divided into 7 groups (*n* = 4) and irradiated at 0, 6, 12, 24, 48, 192 and 384 hours. At the end of the experiment, each sample was investigated via scanning electron microscopy (SEM) and electrochemical measures. Results: The control sample revealed a smooth and compact surface. The small micro-sized porosity is slightly visible at the macroscopic level, but the precise details cannot be observed. A mild exposure to the radioactive solution for 6 to 24 h showed a good preservation of the macro-structural aspects such as thread details and surface quality. Significant changes occurred after 48 h of exposure. It was noticed that the open-circuit potential (OCP) value of the non-irradiated implants move toward more noble potentials during the first 40 min of exposure to the artificial saliva and then stabilizes at a constant value of −143 mV. A displacement of the OCP values toward more negative values was observed for all irradiated implants; these potential shifts are decreasing, as the irradiation period of the tested implants increased. Conclusion: After exposure to I-131, the structure of titanium implants is well preserved up to 12 h. The eroded particles start to appear in the microstructural details after 24 h of exposure and their numbers progressively increase up to 384 h after exposure.

## 1. Introduction

Thyroid cancer is the most frequent endocrine tumor, even if it is considered to be a rare cancer; however, incidences of thyroid cancer are a maximum of 6 cases in 100,000 inhabitants—and this is continuously increasing [1]. According to the 2022 WHO (World Health Organization) classification of thyroid neoplasm, thyroid cancer is classified by its histology in malignant follicular cell-derived neoplasms, stratified based on molecular profiles and aggressiveness, medullary thyroid cancer, anaplastic thyroid cancer and some other rare forms [2]. From the total of thyroid cancers, more than 80% are represented by the malignant follicular cell-derived neoplasms, mostly papillary thyroid cancers [2].

The treatment of the malignant follicular cell-derived neoplasms is established in multidisciplinary committee and is made according to the stage of the disease [3,4]. The first intention procedure in the treatment of the thyroid cancer is the thyroidectomy or the lobectomy, with or without lymphadenectomy [5]. Radioiodine-131 (I-131) therapy is the procedure used for the irradiation of the remnant iodine avid tissue and/or iodine avid metastases using radioiodine (I-131), orally administered [3,5]. Further, hormone therapy for the thyroid function should be administered, using TSH substitutive/suppressing doses of Levothyroxine (LT4), according to the risk of recurrence classification [6].

In the multidisciplinary context of thyroid malignancy, dentistry has a clear role in defining the patients’ quality of life. Oral complications can compromise the protocols of radioiodine I-131 therapy, possibly making it necessary to adjust the administered activities, to change the treatment protocol, or even to avoid the therapy [7,8]. The role of the dental practitioner is to prevent and to erase the oral acute and late side effects of the radioiodine therapy [9]. The effect of radioiodine on salivary glands and its potential inflammatory effect, leading to chronic sialadenitis [9,10,11], is well known; moreover, many publications focus on this side effect and on strategies for preventing its occurrence. Other complications that may arise could be candidiasis, stomatitis, xerostomia, carious lesions, periodontal disease, nerve damage (e.g., facial nerve) or neoplasia [12,13,14,15].

Therapeutic approaches to reduce the infectious risk by treating the carious lesions and removing deficient tooth restorations are essential for preventing periodontal disease [15]. The characteristic of radioiodine to penetrate tooth structure is an important aspect that should be taken into account [15]. Salivary side effects correlate with cumulative radioiodine activity, and thus, dentists managing oral health thyroid patients need to intervene with measures that reduce oral complications. When it comes to oral rehabilitation of thyroid cancer patients with dental implants, the current scientific literature is scarce. Patients with differentiated thyroid cancer during radioiodine therapy are instructed to follow comprehensive rules after taking I-131 for a specific period, as I-131 has a physical half-life of 8 days. The effective half-life in DTC with thyroidectomy is controversial, but many researchers estimate for an activity of 3.7 GBq I-131—the effective half-life in final phase—at 38.6 h [16]. It is rational to consider the complete absence of any radioiodine trace and any direct salivary effect at 10 half-life, approximatively 2 weeks (384 h); this interval could be safe. Therefore, the aim of our study was to characterize the effects of I-131 on the structure on titanium implants up to 384 h after I-131 exposure.

## 2. Materials and Methods

### 2.1. Radioiodine-131 Irradiation Protocol and Sample Preparation

For this study, ethics committee approval was not necessary. Our in vitro model analogue with the in vivo conditions of thyroid cancer patients treated with radioiodine-131 consisted in a solution of 592 MBq (16 mCi) I-131 dissolved in 50 mL of artificial saliva [14,17]. This experimental model was already tested in other in vitro publications by the authors [14,17]. In the prepared solution, titanium implants (*n* = 24) were introduced and were then taken out at 6, 12, 24, 48, 192 and 384 hours post-irradiation [16]. The control dental implants (*n* = 4) were submerged in 20 mL of artificial saliva. Every implant was separately soaked in exactly the same quantity of saliva and I-131 activity. A calibrator (Curiementor 3, Freiburg, Germany) was used to measure the radioactivity of the implants after different exposure times.

### 2.2. Electrochemical Measurements

Electrochemical measurements were conducted in a conventional three-electrode cell using the implant specimens as working electrodes, while a saturated calomel electrode (SCE) and a large Pt foil served as reference and counter electrodes, respectively. For the corrosion testing, the tip of each miniscrew up to a standardized 5 mm level was exposed to the electrolyte solution (artificial saliva), while the remaining part of the implant was carefully coated with Parafilm and insulated from solution by a home-made plastic tube. The electrical connection was established using a copper rod. The surface area of samples exposed to the electrolyte was estimated at 1 cm^2^. A volume of 30 mL of artificial saliva was used for the electrochemical experiments.

Prior to the electrochemical measurement, the dental sample was cleaned with ultrapure water, pure ethanol and dried at room temperature. Then, the implant was left for 2 h in the electrolyte solution to attain the steady-state condition, while measuring the value of open-circuit potential (OCP) as a function of time. Electrochemical impedance spectroscopy (EIS) measurements were acquired at the open-circuit potential using a PARSTAT 2273 potentiostat/galvanostat in the frequency range between 10 kHz and 10 mHz, with 5 points per Hz decade and an AC voltage amplitude of ±10 mV. The fitting of experimental EIS data was performed with ZSimpWin 3.21 software (Ametek, Berwyn, PA, USA). Potentiodynamic polarization measurements were carried out using a Gill AC potentiostat (ACM Instruments, Cartmel, United Kingdom), in the potential range from −300 mV to +3000 mV vs. OCP, with a scan rate of 1 mV/s. The analysis of the polarization results was performed by means of the Gill AC potentiostat software (version 5).

### 2.3. Scanning Electron Microscopy Assessment

Titanium screw samples were rinsed very well with deionized water to remove all traces of radioactive solution exposure and perfectly dried and stored in sterile vials. Their surface was investigated by scanning electron microscopy (SEM) using a Hitachi SU8230 microscope, Hitachi Hi-Tec Corporation, Tokyo, Japan, using an acceleration voltage of 30 kV in high vacuum mode. The macroscopic aspect of the samples was observed at a magnification of ×30, overall microstructure aspect was investigated at ×1000 magnification and microstructural details were observed at a magnification of ×5000.

## 3. Results

### 3.1. Scanning Electron Microscopy Analysis

**Figure 1:**
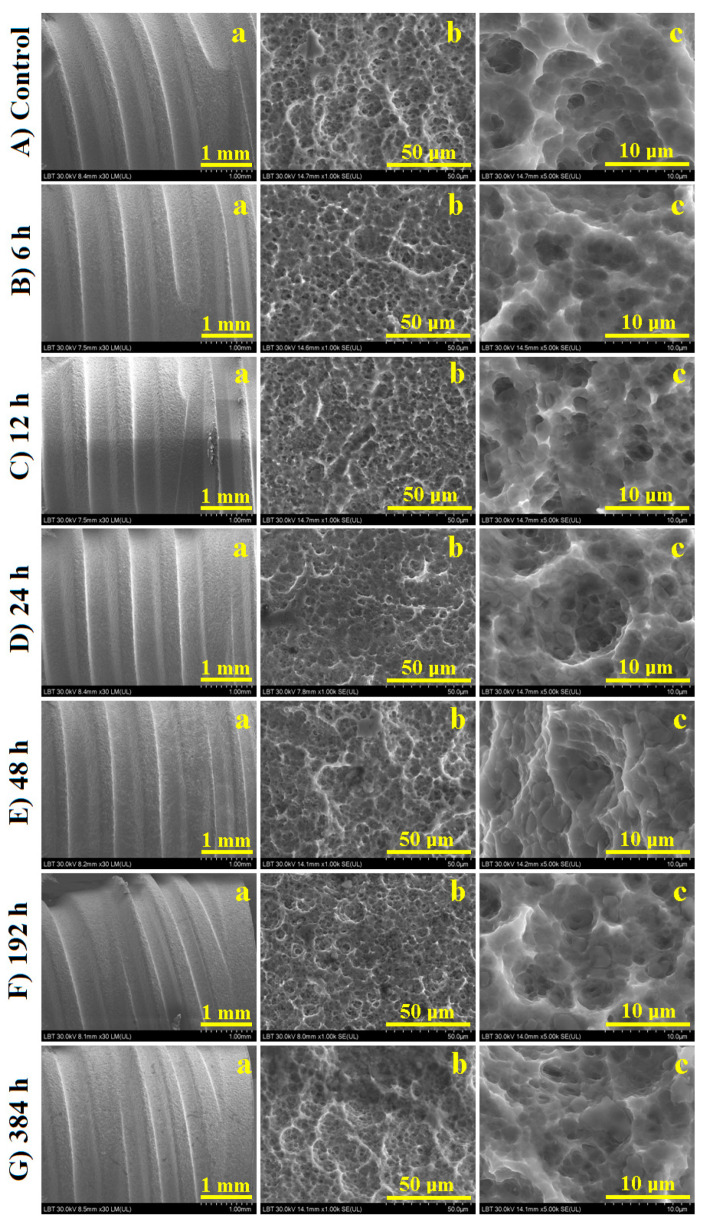
SEM images for Ti screws: macroscopic view (**a**), microstructure (**b**) and fine microstructural details (**c**) exposed to radioactive environment for: (**A**) control, (**B**) 6 h, (**C**) 12 h, (**D**) 24 h, (**E**) 48 h, (**F**) 192 h and (**G**) 384 h.

The control sample macrostructure (Figure 1A(a)) reveals a smooth and compact surface of the thread helix and the constant pitch; moreover, the thread end is observed on the right side of the observation field. The thread root is very smooth and compact; crests are well contoured with a right profile. The small micro-sized porosity is slightly visible at the macroscopic level, but the precise details cannot be observed. A mild exposure to the radioactive solution for 6 to 24 h (Figure 1B(a),C(a),D(a)) shows a good preservation of the macro-structural aspects such as thread details and surface quality.

Significant changes occur after 48 h of exposure (Figure 1E(a)). The thread crest presents some local dimensional alteration prone observed on the right side of the observation field, but the root seems to be well preserved along with the surface porosity which seems to be slightly affected. The thread helix with both crest and root is significantly damaged after 192 and 384 h of exposure to the radioactive solution (Figure 1F(a),G(a)). Surface quality depreciation is also noticed after long-time exposure to the radioactive environment. A more detailed microscopic investigation is required to evidence the specific alterations.

Surface microstructure is very complex due to an interlocked porous structure formed by large alveolus of about 10–20 μm in diameter with smaller pores on their walls of about 2.5 μm diameter (Figure 1A(b)). The pore structure is sustained by a complex network of well sintered titanium particles (e.g., pores walls represent the sintering necks between titanium particles). The microstructural aspects are well preserved at moderate exposure times such as 6 to 12 h (Figure 1B(b),C(b)), with no visible alterations.

Several microstructural alterations start to appear after 24–48 h of exposure that affects the pore walls, which tend to be eroded such as evidenced by the flattened and enlarged pore wall observed in Figure 1D(b),E(b). The prolonged exposure to the I-131 substance at 192 and 384 h facilitated the erosive pattern proliferation (Figure 1F(b),G(b)). The eroded material is dismantled from the pore walls and precipitates inside the pores as boulder particles; their shape and size are well observed at the microstructural details.

### 3.2. Open-Circuit Potential Measurements

**Figure 2:**
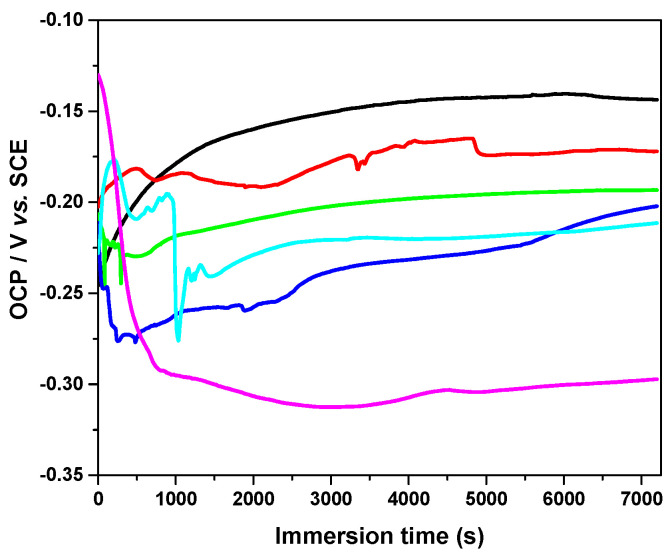
Variation of the open circuit potential of the non-irradiated and irradiated implants exposed for 2 h in artificial saliva solution. Irradiation period (hours): 0 (**—**); 6 (**—**); 12 (**—**); 48 (**—**); 192 (**—**); 384 (**—**).

The evolution of the open-circuit potential (OCP) values for the non-irradiated and irradiated implants during the immersion is shown in Figure 2. In this figure, it can be noticed that the OCP value of the non-irradiated Ti-6Al-4V implant moves toward more noble potentials during the first 40 min of exposure to the artificial saliva and then stabilizes at a constant value of −143 mV vs. SCE. A displacement of the OCP values toward more negative values was observed for all irradiated implants during the immersion in the I-131. These potential shifts are more negative, as the irradiation period of the tested implants increased. For instance, in case of the sample irradiated for 6 h, the OCP value stabilizes at about −172 mV vs. SCE, after 120 min of immersion in artificial saliva. Instead, the open-potential values of the implant irradiated for 384 h drop sharply to a very negative potentials within the first 15 min of immersion and then stabilizes at about −298 V vs. SCE by the end of the exposure period.

### 3.3. Electrochemical Impedance Spectroscopy

The EIS tests were further performed after 2 h exposure of the non-irradiated and irradiated implants in artificial saliva solution and the obtained impedance spectra are presented in Figure 2. The data are displayed in complex impedance (Nyquist diagram) and in Bode amplitude and phase angle plots. As depicted in Figure 2, a single broad capacitive loop is visible in the impedance spectra corresponding to all tested implant specimens exposed for 2 h in the artificial saliva, disregarding their initial condition (non-irradiated or irradiated for different periods). A decrease of the diameter of the capacitive semicircle could be noticed for the irradiated Ti-6Al-4V samples as compared to the untreated one. Likewise, the impedance modulus determined at low frequency (0.01 Hz), |Z|_0.01Hz_ presents smaller values in the case of the treated Ti-6Al-4V implants, with the lowest |Z|_0.01Hz_ values of 214 kΩ cm^2^ and 189 kΩ cm^2^ being attained for the specimens irradiated for 192 h and 384 h, respectively. Moreover, the phase angle maximum in artificial saliva was found to lie in the range of −78° to −70°.

**Figure 3:**
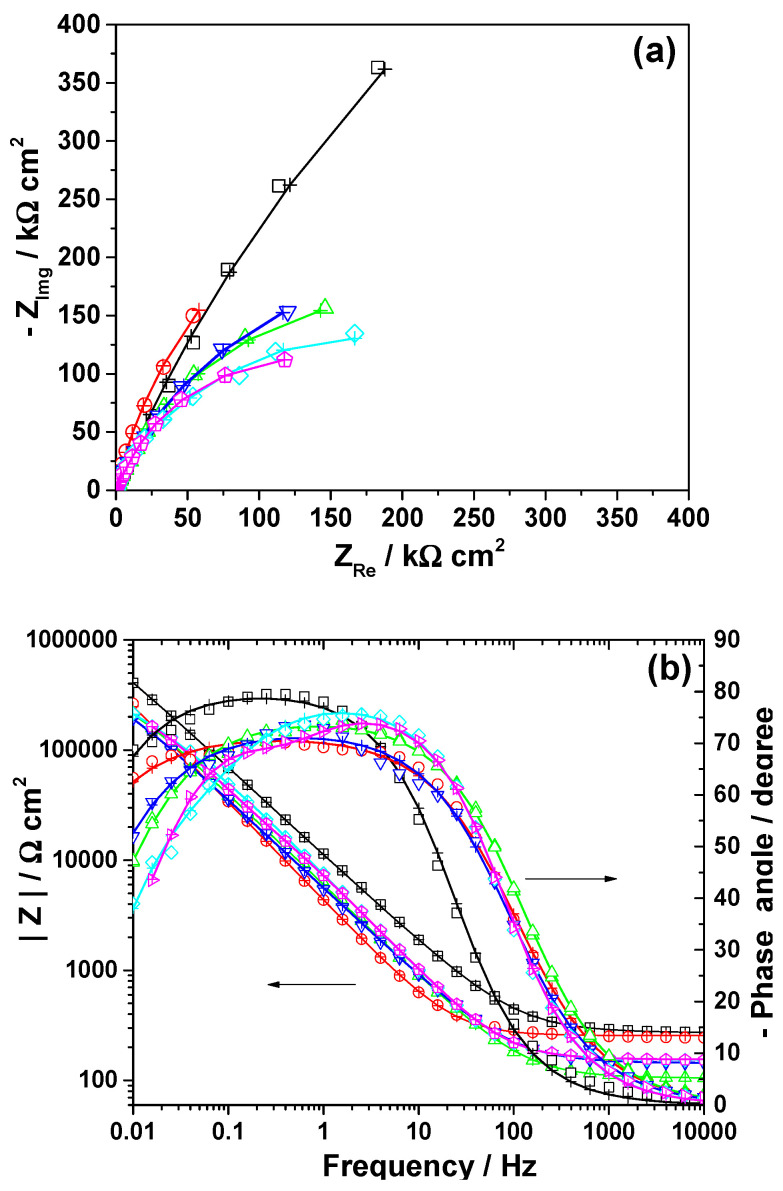
Nyquist (**a**) and Bode (**b**) diagrams corresponding to the non-irradiated (□) and irradiated Ti-6Al-4V implant specimens for 6 h (○); 12 h (∆); 48 h (∇); 192 (◊) and 384 h (⌂) after their exposure for 2 h in the artificial saliva. Symbols correspond to the experimental data and the line with cross (—+—) to the calculated data.

**Figure 4:**
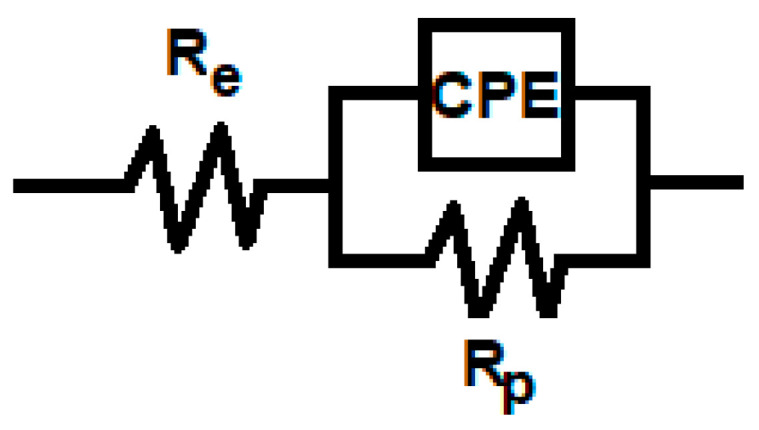
Electrical equivalent circuit used for the computer fitting of experimental data.

As the impedance spectra from Figure 3 consisted of one-time relaxation constant, the equivalent electrical circuit depicted in Figure 4 was further used to reproduce the experimental data and to extract the corresponding R-Q parameters. As depicted in Table 1, the values of the resistance of the passive film are lower in the case of all irradiated samples as compared to the non-irradiated samples. The smallest R_p_ values were calculated for the samples which were irradiated for the longest periods (192 h and 384 h). The Q values for the irradiated samples were lower than those corresponding to the untreated one, while the values of *n* were found within the range of 0.797–0.893, indicating the surface heterogeneity of Ti-6Al-4V samples.

## 4. Discussion

Thyroid cancer is the most frequent endocrine tumor, with specific therapies that succeed in offering complete remission in more than 90% of patients [18]. One of the early side effects of radioiodine used to treat malignant differentiated thyroid disease is acute sialadenitis; the prevalence of salivary dysfunction varies among studies between 16 and 54% [19]. In the majority of cases, sialadenitis resolves within days; however, in some circumstances (high doses, recurrent doses, etc.), there is damage to acinar cells and the inflammatory effect of salivary ducts lead to fluid reduction and chronic sialadenitis, finally ending with xerostomia [19]. The standard protocol to reduce the salivary gland damage is to stimulate the salivary fluid before and after the therapy with lemon juice, and drops for several hours for 2–3 days [9,10].

The quality of life of thyroid cancer patients is an essential issue considering the high rates of curability. Among many other aspects, oral health is an important topic that should be considered in the evaluation of life quality. A previous study performed by our team has evaluated, in vitro, the alteration of enamel and dentin after I-131 exposure using histopathological assessment, scanning electron microscopy (SEM) and atomic force microscopy (AFM) [14]. The results of our research demonstrated that the alterations are extended into the enamel depth after 6 h; furthermore, this is more important after 12 h and the microstructure is modified after 8 days. The next focus was to study if there are any alterations of different types of dental implants in cases of patients with thyroid cancer who underwent radioiodine therapies. The investigated titanium dental implant is produced by powder metallurgy techniques assuring a well-defined surface quality and a precise thread detail, as well as a very well-controlled micro-sized porosity. The microstructural porosity is necessary for the penetration of body fluid, which promotes the hard tissue adhesion onto the implant. According to our latest search in the scientific English language publications database, this is the first study considering the I-131 effects on dental implants.

Complex pore structure nanostructure details within the control sample are observed in Figure 1A(c), with larger alveolus observed and the fine pores observed on their walls. This structure is well preserved after I-131 exposure up to 12 h (Figure 1B(c),C(c)). Eroded particles start to appear in the microstructural details after 24 h of exposure (Figure 1D(c)) as small boulders of about 2 μm which are sank on the pore bottoms. Their diameter strongly increases at about 3–6 μm after 48 h of exposure, as observed in Figure 1E(c), and their number progressively increases up to 192 h of exposure as observed in Figure 1F(c). The longest exposure time of 384 h conducts larger eroded bolder particles of about 8 μm diameter as observed in Figure 1G(c).

The presence of titanium implants in irradiated areas can create a deleterious effect on bone tissue. While studies on external irradiation were previously done, they are completely missing for internal I-131 irradiation. High-energy beta-emitters from I-131—the gamma rays and electrons at the tissue–metal interface—may compromise bone repair. In addition, ionizing radiation induces persistent hypoxia in small blood vessels and decreases the activity and quantity of osteoblasts and osteocytes, which can increase the occurrence of necrosis [20,21]. Implant morphology has a crucial role in bone-implant contact and can enhance the osseointegration process [20,21].

The microscopic alterations on dental implants seen after therapeutic radioiodine exposure have important clinical impact. Firstly, in order to prevent any damage and further dental complication, the patients with thyroid cancer that are candidates for radioiodine therapies will be advised to have their dental implants of titanium after the radioiodine therapy, with a safe period of a minimum of 2 weeks. Secondly, for patients who already have dental implants—and before our clinical evaluation of radioiodine therapy—we need to include data about the type of implants and the clear recommendation to extend the standard salivary stimulation protocol at minimum 2 weeks after irradiation.

## 5. Conclusions

This is the first study considering the effects of the therapeutic I-131 on titanium dental implants. After exposure to radioiodine, the structure of dental implants are well preserved up to 12 h; the eroded particles start to appear in the microstructural details after 24 h of exposure and their number progressively increases up to 384 h after exposure at about 8 μm diameter.

According to our results, there are two major clinical impacts of microscopic alterations on dental implants after radioiodine exposure: (1) in order to prevent any damage and further dental complication, the patients with thyroid cancer that are candidates for radioiodine therapies will be advised to have their dental implants after the radioiodine therapy, with a safe period of a minimum of 2 weeks; and (2) for patients who already have dental implants, we recommend registering their information about the type of dental implants in our clinical files and extending the standard salivary stimulation protocol at minimum 2 weeks after irradiation. Studies on larger cohorts are necessary in order to establish a safe appropriate time.

## Figures and Tables

**Table 1:** EIS parameters for non-irradiated and irradiated implants after 2 h immersion in artificial saliva solution.

Irradiation Period (Hours)	R_e_ (Ω cm^2^)	Rp (kΩ cm^2^)	Q (μF s^n−1^ cm^−2^)	*n*	C (μF cm^−2^)
0	255	1100	44.9	0.893	71.7
6	301	1090	19.5	0.797	57.2
12	105	436.7	36.2	0.833	63.1
48	144	532.7	40.6	0.809	84.1
192	153	295.3	28.7	0.861	37.6
384	152	285.5	30.6	0.848	45.1

## Data Availability

The data presented in this study are available on request from the corresponding author.
